# Common Mind Troubles and the Secret of a Clear Head

**Published:** 1880-04

**Authors:** 


### BIBLIOGRAPHICAL.

COMMON MIND TROUBLES AND THE SECRET OF A CLEAR HEAD. By J. Mortimer Granville, M.D., M.R.C.S., etc. Edited, with additions, by an American Physician, Philadelphia. D. G. BRINTON: 115 South 7th St. 1880.

We have not had the time since the reception of this little book to give an examination sufficient to express any decided opinion of its merits. The subject, however, is an interesting one, and the style, mechanical execution, etc., being good, the work will doubtless prove acceptable.

---

PAMPHLETS RECEIVED.—Gleanings from the History of Medicine. An Address delivered in Evansville, Ind., Nov. 4, 1879. By J. A. Ireland, M.D., of Louisville, Ky., President of the Tri-State Medical Society. Reprint from the *St. Louis Medical and Surgical Journal*, Jan. 5, 1880. St. Louis: Geo. O. Drummond & Co. 1880.

Annual report of the Superintendent of the Cincinnati Sanitarium, for the year ending Nov. 30, 1879. Cincinnati: A. H. Panusford & Co., printers. 1880.

On the Nomenclature and Classification of Diseases of the Skin, with remarks upon that recently adopted by the American Dermatological Association. By L. Duncan Bulkley, A.M., M.D., Physician to the Skin Department Demilt Dispensary, New York, etc., etc. Reprint from Archives of Dermatology, April, 1879.

The Use of Water in the Treatment of Diseases of the Skin. By L. Duncan Bulkley, A.M., M.D., etc., etc. Reprint from *Chicago Medical Journal and Examiner* for January, 1880. New York. 1880.

Electricity in Medicine and Surgery, with cases to illustrate. By John C. Caldwell, M.D., Baltimore, Md. Practice limited to diseases of the nervous system. *Price twenty five cents.*

The Fallacies of Popular Clinical Medicine. By Jarvis S. Wright, M.D., Prof. of Surgery, an introductory lecture delivered at Long Island College Hospital, Brooklyn, N. Y., Feb. 5, 1880. New York: G. P. Putnam's Sons. 1880.

A Plea for Cold Climates in the Treatment of Pulmonary Consumption. Minnesota as a Health Resort. By Talbot Jones, M.D., of St. Paul, Minn. Reprint from *New York Medical Journal*, New York. 1880.

Vicarious Menstruation. By H. G. Landis, A.M., M.D. Reprint from the *Cincinnati Lancet and Clinic*, Feb. 28, 1880.

Excerpta from the Annual Report to the Board of Health for 1879. By Joseph Holt, M.D., Sanitary Inspector of the first district of New Orleans.

Therapeutic Action of Mercury. Inaugural Thesis read before the Chicago Biological Society, Feb. 4, 1880, by S. V. Clevenger, Chicago, Ill. Reprint from the *Chicago Medical Gazette*, Feb. 20, 1880. Chicago: Knight & Leonard, Printers. 1880.

